# Poultry-Like pA+ Biotype of *Staphylococcus aureus* CC346/084 Clone in Human Population

**DOI:** 10.1007/s00284-016-1033-9

**Published:** 2016-04-09

**Authors:** Lidia Piechowicz, Katarzyna Garbacz

**Affiliations:** Department of Medical Microbiology, Medical University of Gdansk, ul. Do Studzienki 38, 80-227 Gdańsk, Poland

## Abstract

The aim of the study was (1) to analyse the prevalence of P-like pA+ biotype of *S. aureus* in material from healthy and diseased individuals, not employed at slaughterhouses or meat processing plants, and (2) to analyse the relatedness of these strains and their genetic variability. The study included 344 strains of *Staphylococcus aureus* isolated from hospitalized patients with staphylococcal infections and from healthy carriers. The biotypes of *S. aureus* were determined on the basis of fibrinolysin and β-haemolysin production, coagulation of bovine plasma, and type of growth on crystal violet agar. Additionally, the strains were tested for the synthesis of protein A in order to distinguish between P-like pA+ and poultry biotypes. Fibrinolysin gene (*sak*) and methicillin resistance (*mecA*) were detected by means of PCR. The clonal structure of studied strains was analysed using pulsed field gel electrophoresis and sequencing of *spa* gene. Finally, the strains were typed with a basic set of 23 bacteriophages. The strains belonging to P-like pA+ biotype corresponded to nearly 20 % of all the studied strains. In contrast to the human biotype, they formed one clonal complex, *spa*-CC346/084. The P-like pA+ biotype strains did not synthesize fibrinolysin, lacked the *sak* gene, and showed susceptibility to methicillin. In contrast to the human biotype strains, they belonged mostly to phage group II. The P-like pA+ biotype strains, previously described solely in meat products and meat industry workers, can be also present in hospitalized patients and extra-hospital carriers. These strains form a single, fibrinolysin-negative, clonal complex t084/CC346.

## Introduction

*Staphylococcus aureus* is an important human and animal pathogen [5, 19, 20, 28, 29]. The strains of this species are divided into a human biotype and animal biotypes [9, 23]. In the 1980s, Devriese proposed to classify human and animal isolates of *S. aureus* on the basis of four biochemical tests (synthesis of fibrinolysin and β-haemolysin, coagulation of bovine plasma, and type of growth on medium containing crystal violet) and phage typing [4, 7]. He identified four biotypes of *S. aureus*: human, bovine, ovine, and poultry; the strains which could not be classified into any of these biotypes on the basis of their properties were referred to as non-host-specific (NHS). In the 1990s, Isigidi and Devriese described a new biotype, P-like pA+ (Poultry-like protein A positive), which showed typical properties of the poultry-biotype but differed from it in terms of the synthesis of protein A [11]. This biotype was detected in meat products and in carriers, employers of slaughterhouses and meat processing plants [11]. Isolation of P-like pA+ from individuals who are not occupationally exposed to meat is an evidence of extremely rare occurrence.

The aim of this study was to analyse the prevalence of P-like pA+ biotype of *S. aureus* in material from healthy and diseased individuals who were not employed at slaughterhouses or meat processing plants. Moreover, we analysed the relatedness of these strains and their genetic variability.

## Methods

### Bacterial Strains

The protocol of the study was approved by the Local Bioethical Committee of the Medical University of Gdansk. We analysed a total of 344 strains of *S. aureus* isolated from hospitalized patients and healthy carriers between 2008 and 2011. The clinical material originated from patients of 10 hospitals, mostly located in Northern Poland. The isolates were taken as part of standard patient care. The samples were obtained from wounds (*n* = 62), pus and purulent lesions (*n* = 45), throats (*n* = 32), blood (*n* = 28), bronchial fluid (*n* = 25), genital tract (*n* = 21), ear (*n* = 19), eye (*n* = 11), and urine (*n* = 9). Additionally, nasal swabs (*n* = 92) were obtained from patients and healthy carriers.

### Identification of Staphylococcal Strains

Specimens were subcultured onto Columbia blood agar and incubated at 35 °C for 24 h. Suspected staphylococcal isolates were identified on the basis of colony characteristics, pigment production, Gram-stained appearance, and haemolysis. The results were confirmed by means of the API ID 32 Staph-system (bioMerieux, Poland) in accordance with the manufacturer’s recommendations.

### Biotyping

The biotypes were checked according to Devriese’s biotyping scheme modified by Isigidi et al. [11] (Table 2). The results of four tests (fibrinolysin production, β-haemolysin production, coagulation of bovine plasma, type of growth on crystal violet agar plates) were used to determine the biotype. Additionally, the strains were tested for the synthesis of protein A in order to distinguish between P-like pA+ and poultry biotypes.

Fibrinolysin production was determined on Nutrient Broth no. 2 with 3 % agar (Nutrient Broth no. 2, Oxoid, UK) [4]. *S. aureus* ATCC 25923 strain was used as a positive control.

For the bovine coagulase production, five drops of an overnight broth culture (Difco, USA) of the staphylococcus were added to tubes containing 0.5 ml of bovine plasma (Sigma, USA) diluted 1–10 in isotonic saline. The tubes were then incubated at 37 °C and examined after 1 and 6 h for the formation of a visible clot [4].

Crystal violet reaction was evaluated by the method of Meyer [18].

Protein A was estimated according to Kerr et al. [13]. *S. aureus* Covan I was used as a positive control.

### Detection of Fibrinolysin Gene (*sak*) by PCR

DNA was isolated according to Barski et al. [1]. The PCR of fibrinolysin gene (*sak*) was performed with the primers SAK-1 and SAK-2 (Sigma, USA) as described by Kim et al. [14]. The PCR product was analysed on 2 % agarose gel (Sigma, USA) in the presence of ethidium bromide under UV illumination. *S. aureus* Wood 46 and *S. aureus* ATCC 25923 were used as a positive control for *sak* gene.

### Detection of Methicillin Resistance

Methicillin resistance was verified by *mecA* gene amplification [1].

### Bacteriophage Typing

Bacteriophage typing was performed using the international set of phages for typing human strains according to the method described by Blair and Williams [2].

### PFGE Typing

Chromosomal grade genomic DNA preparation and SmaI (New England, Biolabs, Beverly Mass.) digestion were carried out as described previously [3]. PFGE was run in a CHEF-DR II system (BioRad, USA). The PFGE results were interpreted according to Tenover et al. [27]. During the classification of the isolates, the samples with identical patterns were considered as representatives of a single PFGE type and were designated with a capital letter; isolates that differed by one to six bands were identified as subtypes of the same type and marked by a supplementary Arabic number.

### *spa* Typing

All PFGE types, including one isolate of each subtype of poultry-like strains (*n* = 23), were subjected to sequencing of PCR product with ABI 377 device (Applied Biosystems, Foster City, CA, USA). The obtained short sequence repeats (SSR) were numbered and processed with the Ridom SpaType software available at http://spaserver.ridom.de. The results were presented as numeric codes corresponding to various *spa* types. To determine clonal relationships between the strains representing various spa types, they were grouped into clonal complexes (*spa*-CC) using BURP algorithm and a demo version of Ridom Staph Type software [17]. The name of a clonal complex (*spa*-CC) originated from the so-called “founder”, i.e. the *spa* type showing the strongest relatedness to all other *spa* types present within the complex. The clonal complex comprised various *spa* types for which the degree of evolution between two strains (“cost”) of different *spa* types was lower than 4 [16, 17].

### Statistical Analysis

The fractions of staphylococcal isolates were presented as number and percentage distributions and compared with Pearson’s Chi-square test and Fischer’s exact test. All calculations were carried out with the Statistica 10 (StatSoft^®^, Tulsa OK, USA) package, with the level of significance set at *P* ≤ 0.05.

## Results

### Detection of Poultry-Like *S. aureus* Strains

Overall, 66 strains of poultry-like biotype were isolated, which corresponded to 19.2 %. There were 47 (18.6 %) poultry-like strains amongst 252 *S. aureus* isolates from pathological lesions; moreover, 19 (20.7 %) such strains were identified amongst healthy carriers. The poultry-like strains were found in 8 out of 10 hospitals. The frequency of occurrence of such strains amongst staphylococci isolated in these hospitals ranged from 8.1 to 82.4 %, including four hospitals where it was equal to approximately 30 % or more (Table 1). The poultry-like staphylococci were found in patients from various wards in each hospital.

**Table 1:** Occurrence of P-like pA+ biotype strains of *S. aureus* in studied hospitals and in a community setting (carriers)

Hospital	Isolated *S. aureus* strains	P-like pA+ biotype
GDA 1	43	5 (11.6 %)
GDA 2	25	7 (28.0 %)
GDA 3	37	3 (8.1 %)
GDA 4	41	5 (12.2 %)
GDA 5	13	4 (30.8 %)
GDA 6	6	2 (33.3 %)
LEB	17	14 (82.4 %)
SLU	57	7 (12.3 %)
KRA	8	0 (0.0 %)
SIE	5	0 (0.0 %)
CAR	92	19 (20.7 %)
Total	344	66 (19.2 %)

CAR—community (carriers)

### Biotyping of *S. aureus* Strains

According to the algorithm proposed by Devriese and Isigidi (Table 2), most of the studied strains represented human biotype (80.2 %), followed by the representatives of P-like A+ biotype (19.2 %), and only two strains (0.6 %) belonging to NHS 4 biotype (Table 3). The strains of human biotype showing C type growth on crystal violet constituted 58 %, and those with A type growth corresponded to 42 %. In contrast to the poultry-like biotype strains which were all fibrinolysin-negative, all human biotype strains synthesized fibrinolysin.

**Table 2:** Devriese’s biotyping scheme modified by Isigidi et al. [10]

Biotype	Staphylokinase	β-haemolysin	Bovine plasma coagulation (6 h)	Crystal violet type^a^	Protein A
Human	+	±	–	A/C	
Bovine	–	+	+	A	
Ovine	–	+	+	C	
Poultry	–	–	–	A	–
Poultry-like	–	–	–	A	+
NHS 1	+	–	+	A	
NHS 2	+	+	+	A	
NHS 3	–	+	–	A	
NHS 4	–	+	–	C	
NHS 5	–	–	–	C	

^a^Type of growth on crystal violet agar: A—yellow, C—purple; NHS—non-host-specific

**Table 3:** Subdivision of investigated human *S. aureus* strains into biotypes according to Devriese and Isigidi scheme [10]

Biotype	Investigated strains
Human	276 (80.2 %)
P-like pA+	66 (19.2 %)
NHS 4	2 (0.6 %)
Bovine	0 (0.0 %)
Ovine	0 (0.0 %)
Poultry	0 (0.0 %)
Total	344 (100.0 %)

### Detection of Staphylokinase Gene (*sak*) by PCR

**Fig. 1:**
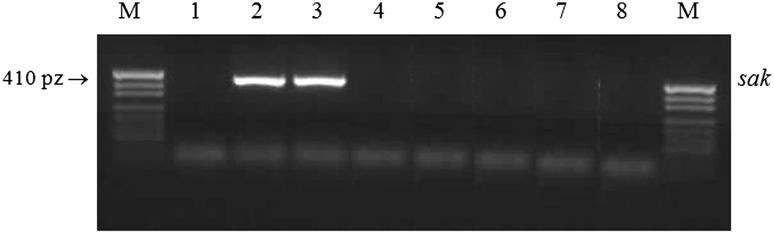
Agarose gel electrophoresis of PCR amplification of fibrinolysin gene (*sak*) product. *M* molecular size marker (pUC19 DNA/MspI enzyme, Fermentas, Lithuania), *lane 1* negative control (PCR mixture), *lanes 2*–*3* positive control (*S. aureus* Wood 46 and *S. aureus* ATCC 25923), *lanes 4*–*8* P-like pA+ *S. aureus* strains

In contrast to the remaining fibrinolysin-positive strains, all strains representing P-like A+ biotype lacked the *sak* gene (Fig. 1).

### Detection of Methicillin Resistance

On the basis of PCR method, all strains P-like A+ biotype were negative for *mecA* gene and were susceptible to methicillin (MSSA).

### Phage Typing of *S. aureus* Strains

The frequencies of poultry-like strains in various bacteriophage groups are presented in Table 4. According to the data, such strains occurred in all the groups, except for group V. Although their prevalence ranged from 1.5 to 4.5 % in the majority of phage groups, it exceeded 84.8 % in group II. Poultry-like biotype strains predominated in phage group II, differing significantly from human biotype strains, prevailing in phage group III (Table 4).

**Table 4:** Distribution of human and P-like pA+ strains of *S. aureus* into various phage groups

Phage group	Human biotype	P-like pA+ biotype	*P* value
Group I	32 (11.6 %)	3 (4.5 %)	0.090
Group II	55 (19.9 %)	56 (84.8 %)	<0.001
Group III	120 (43.5 %)	3 (4.5 %)	<0.001
Group V	15 (5.4 %)	0 (0.0 %)	0.354
Type 95	9 (3.3 %)	1 (1.5 %)	0.449
Mixed group	11 (3.9 %)	2 (3.0 %)	0.715
NT (non-typable)	34 (12.3 %)	1 (1.5 %)	0.009
Total	276 (100.0 %)	66 (100.0 %)	–

### Pulsed Field Gel Electrophoresis (PFGE) Typing

**Fig. 2:**
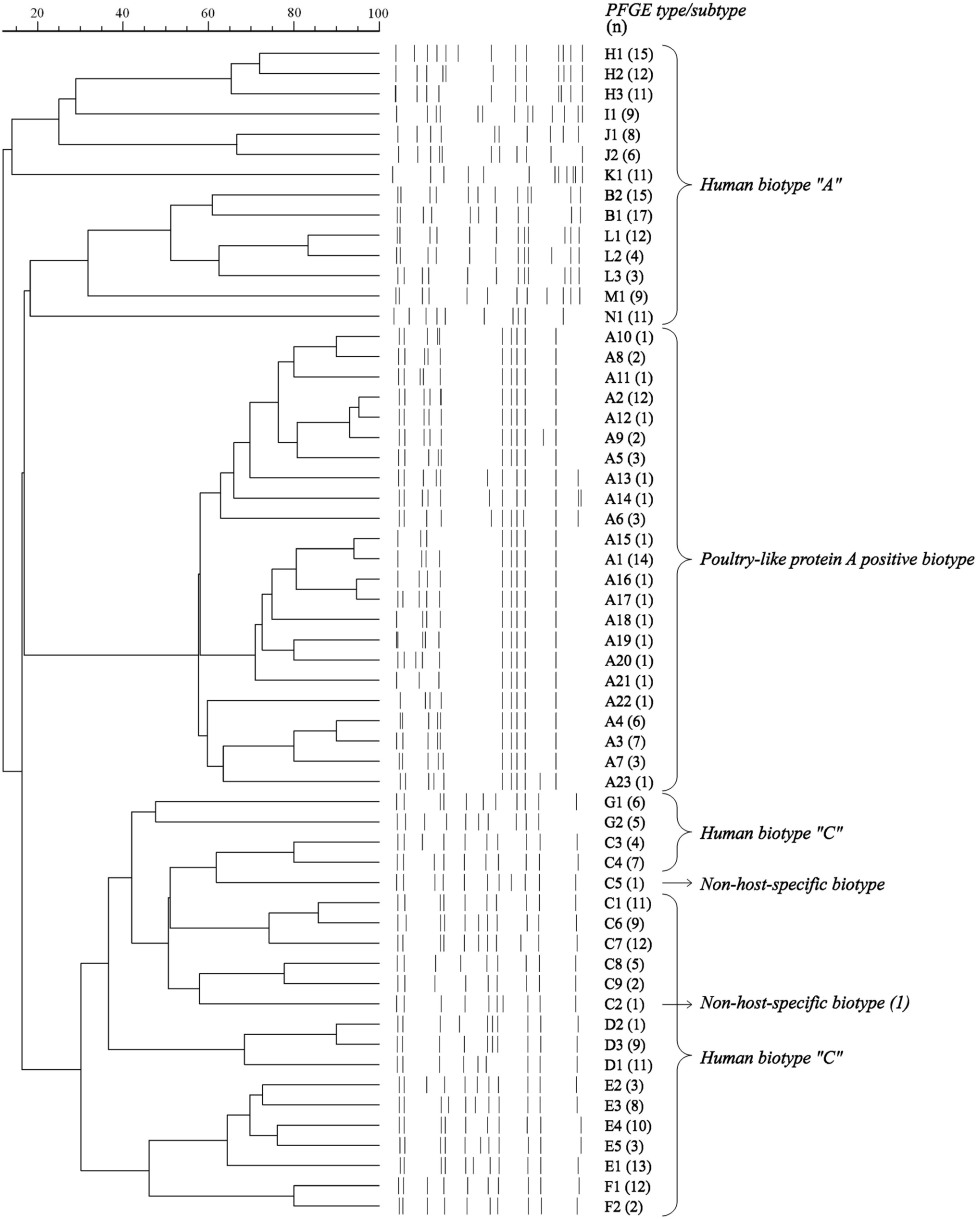
Dendrogram containing PFGE patterns of 344 *S. aureus* strains of different biotypes isolated from humans

The results of the PFGE macrorestriction analysis of the whole chromosomal DNA of studied strains are presented on the corresponding dendrogram (Fig. 2). The analysed population of staphylococci showed marked genetic variability as 14 PFGE types (PFGE-A to PFGE-N) and 58 subtypes were identified. The PFGE-A type comprised a group of P-like pA+ biotype strains (*n* = 66), including 23 various PFGE subtypes (A1-A23) and showing a ≥55 % probability score for clustering in the dendrogram (UPGMA method, Dice coefficient). Based on the criteria proposed by Tenover et al. [27], according to which clone is a group of related isolates belonging to the same PFGE type, we revealed that the population of P-like pA+ staphylococci formed one clone. In contrast, high variability was documented amongst the human biotype staphylococci, which represented 13 PFGE clones and 35 subtypes. The PFGE clones C, D, E, F, and G included type “C” isolates of human biotype, while the PFGE clones B, H, I, J, K, L, M, and N comprised type “A” human biotype staphylococci. The two strains representing NSH4 biotype were characterized by C5 and C2 patterns. Noticeably, none of the human biotype strains belonged to the clone represented by P-like pA+ isolates.

### *spa* Typing

The *spa* gene was sequenced in 40 samples selected from amongst the most prevalent PFGE types and subtypes, including all P-like pA+ biotype strains. The results of the sequencing are summarized in Table 5. Overall, 17 types of *spa* were identified, including one new type not presently listed in the respective database (http://www.ridom.de/spaserver). The P-like pA+ biotype strains included five *spa* types: t084 (18.2 %) was the most prevalent, followed by t346, t254, t144, and t774. We used BURP algorithm to determine clonal relatedness of the identified *spa* types. Two clonal complexes, *spa*-CC346/084 and *spa*-CC435, were identified, along with a group of singletons. The *spa*-CC346/084 complex included solely the P-like pA+ biotype strains of PFGE-A clone. In contrast, the *spa*-CC435 complex included isolates of five other PFGE clones (C, D, E, F, and G) of human biotype. The group of singletons included isolates which did not show clonal relatedness between the *spa* types (*spa*-CC) and represented the remaining PFGE clones (B, H, I, J, K, L, M, and N).

**Table 5:** Spa types and clonal complexes (*spa*-CC) amongst selected PFGE types/subtypes

PFGE subtype	*n*	*spa* type	*spa*-CC
A1, A2, A3, A7, A11, A12, A14, A16, A19, A20, A22, A23	12	t084	CC346/084
A5, A8, A13, A17, A18	5	t346	
A9, A15	2	t254	
A6	1	t144	
A4, A10, A21	3	t774	
C1	1	t435	CC435
C2	1	t4527	
G1	1	t2086	
F1	1	t284	
E1	1	t159	
D1	1	t435	
H1, H2, H3	3	t008	Singleton 1
J1	1	t056	Singleton 2
N1, M1	2	t164	Singleton 3
K1	1	t189	Singleton 4
B1, B2, L1	3	t209	Singleton 5
I1	1	new	Singleton 6

*spa* types available at http://www.ridom.de/spaserver

## Discussion

According to the classification proposed by Devriese and Isigidi, nearly 20 % of staphylococci isolated from participants of our study represented P-like pA+ (Poultry-like protein A positive) biotype. Aside from the lack of fibrinolysin, this biotype is characterized also by the lack of β-haemolysin production, negative reaction with bovine plasma, ability to synthesize protein A, and the “A” type of growth on crystal violet agar [4, 11]. The occurrence of P-like pA+ biotype of staphylococci is surprising in the hospital setting as to date they were mostly isolated from meat products or individuals having contact with animals or products of animal origin [10–12].

The P-like pA+ biotype staphylococci isolated from our hospitalized patients have not been described in the hospital setting thus far. Previous knowledge on P-like pA+ group is limited and existing evidence does not support the presence of these microorganisms in human clinical material [11]. This belief has to be revised in view of our findings.

Macrorestriction analysis (PFGE) revealed one PFGE-A clone within P-like pA+ biotype and sequencing of *spa* gene showed the presence of related strains forming one clonal complex *spa*-CC346/084. Contrary to P-like pA+ biotype, the staphylococci of human biotype showed greater genetic variability. They belonged to several PFGE clones, and only some of them formed one clonal complex, *spa*-CC (CC435). Moreover, none of the human strains belonged to the clone represented by P-like pA+ strains. This confirms that P-like pA+ staphylococci constitute a population which is genetically distinct from other staphylococci isolated from humans. To the best of our knowledge, our paper is the first to address the questions of prevalence and clonal structure of P-like pA+ biotype strains originating from human clinical material. A small number of previous studies dealing with the problem in question referred to humans exposed to contact with animal products [10, 25]. Hennekinne et al. [10] detected P-like pA+ biotype strains representing one PFGE complex in meat products and amongst meat processing workers; similarly, Rodgers et al. [25] examined P-like pA+ strains originating from carriers employed at a poultry hatching farm.

Our findings raise the question on the origin of strains belonging to P-like pA+ biotype. According to literature, to date, the strains of P-like pA+ biotype were sporadically isolated solely from specific environment of slaughterhouses and meat plants, where the employees were exposed to contact with meat or animals [11]. Our study is the first to show the prevalence of this clone of P-like pA+ staphylococci in the hospital setting and in community carriers. There are two possible theories on the origin of the representatives of P-like pA+ biotype in these environments. They can represent animal strains of *S. aureus* that acquired the ability to synthesize protein A, or human staphylococci that lost the ability to produce fibrinolysin [10, 11]. The ability to synthesize fibrinolysin is the principal difference between human and animal strains of *S. aureus* [5, 15]. Our study revealed lack of fibrinolysin synthesis in all strains of P-like pA+ biotype. Moreover, we documented that this resulted from the loss of fibrinolysin encoding gene (*sak*).

In view of the above-mentioned findings, it is most likely that P-like pA+ biotype staphylococci isolated from hospitalized patients and community carriers are the strains which lost the ability to synthesize fibrinolysin. A small number of authors have claimed that staphylococci can show lability of this latter feature [6]. The ability of *S. aureus* to synthesize fibrinolysin is determined by the lysogenic conversion of a bacteriophage containing *sak* gene [21, 30]. Previous studies revealed that fibrinolysin-positive staphylococci of human biotype can lose the ability to synthesize fibrinolysin as a result of adaptation to a new host [6].

The *spa* t084 type, which predominated in the detected clonal complex, also suggests human origin of isolated representatives of P-like pA+ biotype. According to available literature, the t084 type is prevalent amongst *S. aureus* strains of human origin [8, 22, 24]. These data suggest that the representatives of the *spa* t084 type are mostly spread in a population of human strains, and possibly point to human origin of P-like pA+ biotype staphylococci isolated from our participants. Similar conclusions can be drawn on the basis of typing with a set of 23 phages for human strain. All P-like pA+ biotype staphylococci were susceptible to group II phages at concentration equal to 1× RTD. Previous studies showed that animal strains can rarely be typed with phages from basic set for human strains, in contrast to phages for animal staphylococci [26].

On the other hand, it cannot be excluded that the hereby described P-isolates were animal strains that acquired the ability to synthesize protein A. As widely known, colonization of various hosts by staphylococci requires adaptive changes, which in turn can be reflected by acquisition of new biochemical characteristics, such as synthesis of protein A. Consequently, the relatedness of the P-like pA+ isolates from humans and animals still remains an open issue. Although answering this question would expand our knowledge on human–animal transmission of staphylococci, further research is needed to address the problem in question.

In conclusion, this study revealed that the P-like pA+ biotype strains, previously described solely in meat products and meat industry workers, can be also present in hospitalized patients and extra-hospital carriers. These strains form a single, fibrinolysin-negative, clonal complex t084/CC346.
